# Non Alcoholic Fatty Liver Disease Is Positively Associated with Increased Glycated Haemoglobin Levels in Subjects without Diabetes

**DOI:** 10.3390/jcm10081695

**Published:** 2021-04-15

**Authors:** Roberta Zupo, Fabio Castellana, Francesco Panza, Marco Castellana, Luisa Lampignano, Raffaele Ivan Cincione, Vincenzo Triggiani, Gianluigi Giannelli, Vittorio Dibello, Rodolfo Sardone, Giovanni De Pergola

**Affiliations:** 1 Unit of Research Methodology and Data Sciences for Population Health, National Institute of Gastroenterology “Saverio de Bellis”, Research Hospital, Castellana Grotte, 70013 Bari, Italy; fabio.castellana@irccsdebellis.it (F.C.); f_panza@hotmail.com (F.P.); mcastellana01@yahoo.it (M.C.); luisalampignano@gmail.com (L.L.); rodolfo.sardone@irccsdebellis.it (R.S.); giovanni.depergola@irccsdebellis.it (G.D.P.); 2 Department of Clinical and Experimental Medicine, University of Foggia, 71122 Foggia, Italy; ivan.cincione@unifg.it; 3 Section of Internal Medicine, Geriatrics, Endocrinology and Rare Disease, Interdisciplinary Department of Medicine, School of Medicine, University of Bari, 70124 Bari, Italy; vincenzo.triggiani@uniba.it; 4 Scientific Direction, National Institute of Gastroenterology “Saverio de Bellis”, Research Hospital, Castellana Grotte, 70013 Bari, Italy; gianluigi.giannelli@irccsdebellis.it; 5 Department of Orofacial Pain and Dysfunction, Academic Centre for Dentistry Amsterdam (ACTA), University of Amsterdam and Vrije Universiteit Amsterdam, 1081 Amsterdam, The Netherlands; vittoriodibello1@gmail.com; 6 Department of Biomedical Science and Human Oncology, School of Medicine, Policlinico, University of Bari, 70124 Bari, Italy

**Keywords:** non-alcoholic fatty liver disease, glycated haemoglobin, obesity

## Abstract

Screening for non-alcoholic fatty liver disease (NAFLD) is key step for primary management of fatty liver in the clinical setting. Excess weight subjects carry a greater metabolic risk even before exhibiting pathological patterns, including diabetes. We characterized the cross-sectional relationship between routine circulating biomarkers and NAFLD in a large sample of diabetes-free subjects with overweight or obesity, to elucidate any independent relationship. A population sample of 1232 consecutive subjects with a body mass index of at least 25 kg/m^2^, not receiving any drug or supplemental therapy, was studied. Clinical data and routine biochemistry were analyzed. NAFLD was defined using the validated fatty liver index (FLI), classifying subjects with a score ≥ 60% as at high risk. Due to extreme skewing of variables of interest, resampling matching for age and sex was performed. Our study population was characterized by a majority of females (69.90%) and a prevalence of NAFLD in males (88.90%). As a first step, propensity score matching was explicitly performed to balance the two groups according to the FLI cut-off. Based on the resulting statistical trajectories, corroborated even after data matching, we built two logistic regression models on the matched population (*N* = 732) to verify any independent association. We found that each unit increase of FT3 implicated a 50% increased risk of NAFLD (OR 1.506, 95%CI 1.064 to 2.131). When including glycated haemoglobin (HbA1c) in the model, free-triiodothyronine (FT3) lost significance (OR 1.557, 95%CI 0.784 to 3.089) while each unit increase in HbA1c (%) indicated a significantly greater NAFLD risk, by almost two-fold (OR 2.32, 95%CI 1.193 to 4.512). Glucose metabolism dominates a key pathway along the hazard trajectories of NAFLD, turned out to be key biomarker in monitoring the risk of fatty liver in diabetes-free overweight subjects. Each unit increase in HbA1c (%) indicated a significantly greater NAFLD risk, by almost two-fold, in our study.

## 1. Introduction

Non-alcoholic fatty liver disease (NAFLD) is the most common metabolic liver disease, in the form of steatosis not attributable to secondary causes of liver fat accumulation (e.g., significant alcohol consumption, viral infections, medications). The latest reports point out the steadily increasing prevalence of NAFLD worldwide, alongside pandemic obesity and diabetes, affecting 20% of the general population and up to 70% of the diabetic population [1,2]. As so far recorded, NAFLD phenotypes cover a broad spectrum of liver disease, ranging from basic steatosis to steatohepatitis (NASH) or fibrosis, potentially leading to cirrhosis and, at worst, hepatocarcinoma [3]. Nearly 80% of patients with NAFLD are carriers of a morbid obesity phenotype featuring visceral ectopic fat accumulation (4); this feature contributes to a heightened risk of liver cirrhosis [4].

From a metabolic perspective, NAFLD triggers a cascade encompassing impaired insulin sensitivity and glucose homeostasis, and higher levels of liver enzymes, mainly alanine amino transferase (ALT) and gamma-glutamyl transpeptidase (γGT), may predict the onset of type 2 diabetes [5]. The interplay between glucose tolerance, diabetes, and the risk of developing NAFLD is well-documented [6], and this evidence may suggest a common pathophysiological pathway. In this context, the haemoglobin glycation rate is a key indicator of blood glucose concentrations, featuring the unique retrospective capacity to reflect the average glucose concentration over the previous 8–12 weeks. A recent study of apparently healthy individuals showed high glycated haemoglobin (HbA1c) levels to be independently associated with a greater prevalence of NAFLD and an increased risk of advanced fibrosis in NAFLD patients without diabetes [7]. A European study of 143 overweight or obese non-smokers without diabetes showed that NAFLD was best predicted by a combination of age, sex, waist circumference, ALT, insulin resistance, and HbA1c [8].

From the same metabolic perspective, thyroid dysfunction has been documented to be involved in the pathogenesis of NAFLD, perhaps based on the well-established biological role of the thyroid in regulating lipid metabolism, body weight, and insulin resistance [9,10,11]. Accordingly, the prevalence of NAFLD has been found to be consistently higher among patients with primary hypothyroidism, including those with a subclinical phenotype characterized by increased levels of thyroid-stimulating hormone (TSH) but normal free thyroxine (FT4) [12]. Strictly speaking, hypothyroidism is per se associated with dyslipidaemia and obesity, both of which promote the progression of NAFLD [12] with a 1.24-fold increased risk of NAFLD compared to euthyroidism [13].

Despite the large body of evidence on this topic, further studies are needed to confirm any causal relationship between key metabolic risk parameters and NAFLD in subjects with an excess weight phenotype, regardless of potential confounding factors associated with NAFLD. From this standpoint, an interesting confounding factor associated with NAFLD is the glucose metabolism. This study aimed to evaluate the relationship between major routine fluid biomarkers and NAFLD, in terms of effect modification, among 1232 subjects with overweight or obesity but without diabetes.

## 2. Materials and Methods

### 2.1. Study Population and Design

From January 2018 to December 2020, 1232 consecutive patients (861 females, 371 males) were recruited at the “Population Health Unit” of the National Institute of Gastroenterology “S. de Bellis,” Research Hospital (Castellana Grotte, Apulia, Italy). All data were collected at the baseline examination. Inclusion criteria were overweight or obesity (BMI ≥ 25 kg/m^2^) in subjects taking no supplements or medication, including oral contraceptives or medicines for osteoporosis. Exclusion criteria were any history of endocrinological diseases (i.e., diabetes mellitus, hypo or hyperthyroidism, hypopituitarism), chronic inflammatory diseases, stable hypertension, HBV or HCV infections, significant alcohol intake, angina pectoris, stroke, transient ischaemic attack, atrial fibrillation, heart infarction, congenital heart disease, any malignancies, renal or liver failure, and inherited thrombocytopoenia. The study protocol (ClinicalTrials.gov Identifier: NCT04327375) met the principles in the Declaration of Helsinki. All subjects gave their informed consent for inclusion before they participated in the study. The study was conducted in accordance with the Declaration of Helsinki, and the protocol was approved by the Ethics Committee of the National Cancer Institute IRCCS “Giovanni Paolo II” (Project identification code Prot. N. 439/2020).

### 2.2. Clinical Examination and Fluid Biomarkers Collection

At baseline, hormonal, metabolic, and routine biochemistry parameters were closely examined in all subjects. A brief interview, including questions on medical history and lifestyle, was conducted by a senior physician. Extemporaneous outpatients diastolic (DBP) and systolic blood pressure (SBP) were determined in a sitting position after at least a 10-min rest, a minimum of three different times, using an M6 Automatic Blood Pressure monitor (OMRON, Kusatsu, Ayabe, The Netherlands). A smoking habit was also investigated as a dichotomous variable (yes/no). Blood samples were drawn at 08.00–09.00 am, after overnight fasting. Fasting plasma glucose (FPG), HbA1c, insulin, total cholesterol, high-density lipoprotein (HDL) cholesterol, triglycerides, 25OH vitamin D, creatinine, uric acid, and liver markers serum levels were assayed. Serum insulin concentrations were measured by radioimmunoassay (Behring, Scoppito, Italy), and all samples were analyzed in duplicate. Fasting plasma glucose was determined using the glucose oxidase method (Sclavus, Siena, Italy), while the concentrations of plasma lipids (triglycerides, total cholesterol, HDL cholesterol) were quantified by an automated colorimetric method (Hitachi; Boehringer Mannheim, Mannheim, Germany). TSH, FT3, and FT4 serum concentrations were measured by a competitive luminometric assay based on the SPALT (solid-phase antigen luminescence technique) principle (LIAISON FT3, FT4, TSH, DiaSorin, Saluggia, Italy). HbA1c was routinely assayed on a chemical analyzer Architect c8000 (Abbott Laboratories, Irving, TX, USA) Serum 25(OH) vitamin D was quantified by a chemiluminescence method (Diasorin Inc, Stillwater, OK, USA), and all samples were analyzed in duplicate. Serum uric acid was measured by the URICASE/POD method implemented in an autoanalyzer (Boehringer Mannheim). Creatinine was measured by an automated system (UniCel Integrated Workstations DxC 660i, Beckman Coulter, Fullerton, CA, USA). Amino transferase and γ-glutamyl transpeptidase (γGT) were measured with standard routine laboratory methods. Low-density lipoprotein (LDL) cholesterol was calculated using the Friedewald equation [13]. Insulin resistance was assessed using the homeostasis model assessment—insulin resistance (HOMA-IR) [14].

### 2.3. Anthropometric Assessment

Two qualified nutritionists (RZ, LL), trained for equivalent measuring performances, carried out clinical procedures. All anthropometric measurements were taken with participants dressed in lightweight clothing and without shoes. Variables were all collected simultaneously at 7.00–10.00 am, after overnight fasting. Height was measured to the nearest 0.5 cm using a wall-mounted stadiometer (Seca 711; Seca, Hamburg, Germany). Body weight was determined to the nearest 0.1 kg using a calibrated balance beam scale (Seca 711). BMI was calculated by dividing body weight (Kg) by the square of height (m^2^) and classified according to World Health Organization criteria for normal weight (18.5–24.9 kg/m^2^), overweight (25.0–29.9 kg/m^2^), grade I obesity (30.0–34.9 kg/m^2^), grade II obesity (35.0–39.9 kg/m^2^), and grade III obesity (≥40.0 kg/m^2^) [15]. Waist circumference (WC) was measured at the narrowest part of the abdomen or in the area between the tenth rib and the iliac crest (minimum circumference).

### 2.4. NAFLD Assessment

The FLI, a modelling algorithm including BMI, WC, triglycerides, and γGT [16], was used to assess the risk of NAFLD. The calculation was made according to the following equation: (e 0.953 × loge (TG) + 0.139 × BMI + 0.718 × loge (GGT) + 0.053 × WC − 15.745)/(1 + e 0.953 × loge (TG) + 0.139 × BMI + 0.718 × loge (GGT) + 0.053 × WC − 15.745) × 100. Subjects with FLI < 30 are classified as at low risk of NAFLD, and those with FLI ≥ 60 at high risk.

### 2.5. Statistics

We performed statistical analysis of baseline variables, expressed as mean ± Standard Deviation (SD), median and range for continuous variables, and proportion (%) for the frequency of categorical variables. The normality of distribution was assessed for each variable using Shapiro’s test. Spearman’s correlation matrix was built for all continuous biochemical and anthropometric variables to check for interrelated variables to avoid collinearity effects in the model. *p*-values less than or equal to 0.05 were considered statistically significant, with 95% confidence intervals.

To balance the group comparison, propensity score using regression algorithm resampling and nearest neighbor (NN) matching for age and sex was operated. Patients in the high risk group (FLI ≥ 60) were compared with those in the low-to intermediate risk group (FLI < 60) using NN matching for main confounding covariates, i.e., age and sex. Comparative analyses were carried out using non-parametric two-tailed tests.

Following matching, two linear regression models on FLI ≥ 60 pathological status were built to investigate both the exposure risk due to higher FT3 circulating levels and possible independent relationships, according to a hierarchical method: (1) raw model using only FT3 as covariate (2) model 1 plus HbA1c. The methodological approach and analyses were designed and operated by a senior epidemiologist (RS) and biostatistician (FC) using RStudio software, version 1.2.5042.

## 3. Results

The whole sample (*N* = 1232) featured a majority of women (69.90%, *N* = 861 vs. 30.10%, *N* = 371) while, according to the referenced cut-off value, a high risk of NAFLD was found prevalently in males (88.90%, *N* = 330). A comparative descriptive analysis of the sample by NAFLD status and FLI score is shown in Table 1. The high risk group (FLI ≥ 60) featured greater age, BMI, waist circumference, and higher extemporaneous blood pressure (SBP, DBP) values (*p* < 0.019). A smoking habit was also found to prevail among these subjects (*p* = 0.03) as well as, in terms of the metabolic profile, a poor glycaemic and lipid balance, revealing a metabolic syndrome pattern (*p* < 0.01), with significantly lower 25(OH)vitamin D and higher uric acid serum levels (*p* < 0.01). No statistically significant differences in thyroid hormone levels emerged.

**Figure 1:**
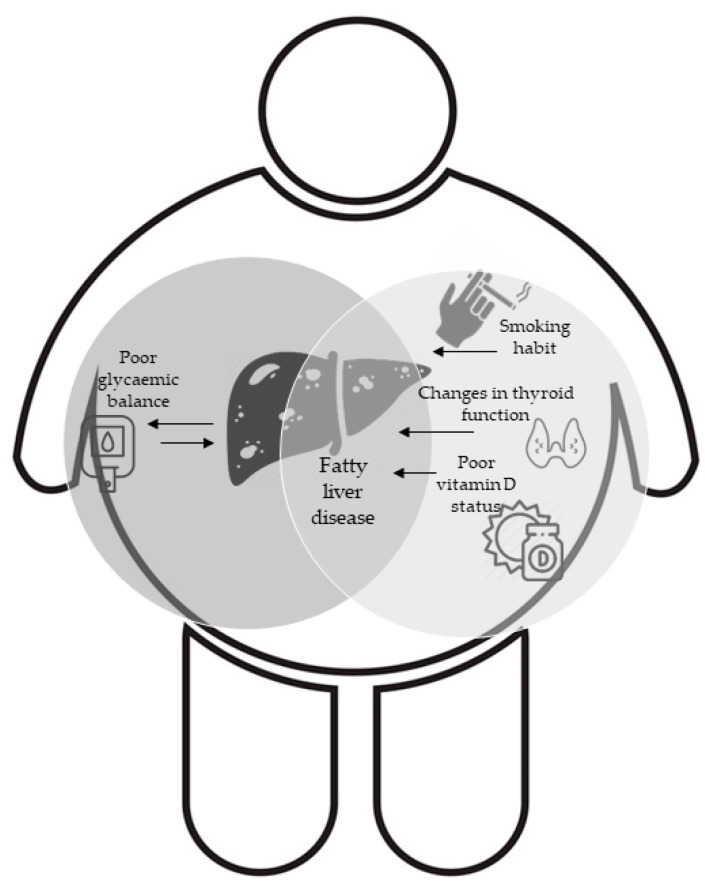
Graphic representation of the interplay between metabolic drivers and Non-Alcoholic Fatty Liver Disease (NAFLD) in obesity.

Applying a propensity score model, we matched 1:1 to obtain two balanced groups (50% FLI < 60 vs. 50% FLI ≥ 60). Thus, an additional comparative analysis was conducted in 732 subjects after the propensity score matching, as shown in Table 2. The matching was based on major confounding covariates, as previously described in the Method section. All between-group discrepancies were corroborated after the matching, except for 25(OH) vitamin D (*p* = 0.08), FT3 serum levels (*p* = 0.04). Figure 1 shows a graphic representation of main findings.

To further assess the association between NAFLD and FT3 ceteris paribus of the major covariates, age and sex, we built two logistic regression models on the matched population (*N* = 732) to verify any independent association (Table 3). We found that each unit increase of FT3 implicated a 50% increased risk of NAFLD (OR 1.506, 95%CI 1.064 to 2.131). When including HbA1c in the model, FT3 lost significance (OR 1.557, 95%CI 0.784 to 3.089) while each unit increase in HbA1c (%) indicated a significantly greater NAFLD risk, by almost two-fold (OR 2.32, 95%CI 1.193 to 4.512).

## 4. Discussion

This study analyzed a large population of diabetes-free subjects with overweight and obesity, providing evidence of a close, positive independent cross-sectional relationship between circulating HbA1c levels and the presence of NAFLD. This survey adds a pending concept within fluid biomarkers of hazard for NAFLD, supported by a score matching methodology applied to the present study sample to strengthen the finding.

As a first finding arising from our analyses and corroborated after matching, the NAFLD prevalence in smokers must be emphasized. This association is consistent with previous longitudinal findings demonstrating a positive association of current smoking, pack-years, and urinary cotinine levels with the risk of incident NAFLD in healthy young and middle-aged subjects [17], and suggests that smoking independently contributes to the development of NAFLD and fibrosis. Among the hypothesized causative pathways, smoking may significantly induce insulin resistance, hyperinsulinemia, dyslipidemia, and hepatic steatosis by altering the lipid metabolism [18,19,20]. Another potential mechanism is the possibility that smoking may induce pro-inflammatory effects and chronic hypoxia, which could play a role in the NAFLD pathogenesis and progression [21]. Also, nicotine may stimulate sympathetic nerve pathways, increasing the release of catecholamines and glucagon, thus contributing to the NAFLD pathogenesis [22,23].

The lower 25(OH)vitamin D levels found in the NAFLD group before matching are concordant with previous reports showing decreased 25(OH)vitamin D concentrations in obese phenotypes [24,25], suggesting that vitamin D deficiency may be involved in the pathogenesis of NAFLD [26]. However, after matching, the statistical difference between groups was lost, showing that sex and age have a significant role in this association.

Consistent with literature data, subjects with NAFLD showed a poor glycaemic balance featuring higher levels of HbA1c, which is a well-known gold standard in tracking the trajectories of glucose homeostasis [27], both before and after the matching. This finding mirrors the acknowledged relationship between NAFLD and insulin resistance [28], supporting the body of research reporting an independent association between HbA1c levels and the NAFLD prevalence in subjects without diabetes [7,8]. Multiple pathophysiologically relevant connections have been postulated to explain this association [29]. In particular, analyses of disease-gene relationship data showed that most of the genes associated with HbA1c levels were also implicated in NAFLD, with a significant overlap. Genes belonging to this overlap are also likely involved in pathways critical to responses to nutrient levels and hormonal stimulation [29].

As regards any possible relationship between other routine metabolic fluid biomarkers and fatty liver, we found no differences in TSH, FT3, and FT4 serum levels between the two groups. After the matching, only FT3 levels were found to be significantly higher in the group at high risk of NAFLD. A positive relationship between NAFLD and FT3 serum levels has previously been demonstrated in two population-based cohort studies of euthyroid subjects [30,31], and recently corroborated in a similar cross-sectional report on morbid obese subjects [32]. However, these reports did not consider glucose homeostasis or autoimmune factors. Our logistic regression models run on the matched population firstly showed a 50% increased risk of NAFLD for each unit increase of FT3 (OR 1.506, 95%CI 1.064 to 2.131), but this finding lost significance when including HbA1c. Each unit increase in HbA1c (%) in our models indicated a significantly greater NAFLD risk, by almost two-fold (OR 2.32, 95%CI 1.193 to 4.512). Our findings failed to demonstrate any direct independent association between FT3 and NAFLD in euthyroid subjects, not confirming the postulated pathogenesis of NAFLD as hypothyroidism-induced in obese subjects.

An important consideration about the effect modification explored in this study is that NAFLD and thyroid functions could both be considered as effects of glycaemic metabolism disorders. This concept fits the findings in a recent review article by Xia and colleagues [33], underlining that impaired glycaemic control and systemic insulin resistance may promote, even before the onset of diabetes, an increase of free fatty acid flux from peripheral tissues to the liver, leading to the development and progression of NAFLD. Furthermore, a glycaemic imbalance may well drive the progression of NAFLD from simple steatosis to non-alcoholic steatohepatitis (NASH), cirrhosis, and hepatocellular carcinoma, through multiple mechanisms including direct hepatocyte lipotoxicity and hepatocellular oxidative stress. The same study postulated that NAFLD and diabetes could be different sides of the same coin, since NAFLD is also involved in the development of diabetes, by increasing glucose production in the liver and exacerbating hepatic insulin resistance through the activation of hepatic protein kinase and some liver-secreted proteins with diabetogenic properties.

Some study limitations should be considered. Due to the cross-sectional nature of the data, we could not assess the temporal nature of associations. If confirmed that HbA1c could cause both NAFLD and a glycaemic disorder, HbA1c should be considered as a collider [34] more than an effect modifier in the causal pathway. Prospective studies are needed to clarify a causal relationship. Yet, both the lack of the imaging for NAFLD evaluation and the missing inflammatory profile contribute to weakening our data. The strong point is that we examined only individuals taking no medication, thus avoiding a possible interference with biomarkers assays and investigational outcomes.

## 5. Conclusions

We conclude that a glycaemic imbalance can both cause and be caused by NAFLD, but longitudinal studies are needed to clarify the precise causal relationship. Furthermore, our study stresses the concept that routine biomarkers of glucose metabolism work better in monitoring the risk pathway of NAFLD in clinical settings, at least in subjects with baseline overweight and obesity.

## Figures and Tables

**Table 1:** Description of the whole sample according to Fatty Liver Index score (*N* = 1232). All data are shown as mean ± SD (min to max) for continuous variables and as (%) for proportions.

Prop. (%)	FLI < 60	FLI ≥ 60	*p* Value *
	366 (29.70)	866 (70.30)	
Age (years)	37.63 ± 12.48	40.11 ± 12.74	**<0.01**
Sex			
*Females*	325 (37.70)	536 (62.30)	**<0.01** **^χ2^**
*Males*	41 (11.10)	330 (88.90)	
BMI (kg/m^2^)	28.85 ± 2.49	36.06 ± 5.91	**<0.01**
Smoking (Yes)	60 (16.90)	189 (22.70)	**0.03** **^χ2^**
Fatty Liver index (%)	37.41 ± 14.62	85.1 ± 11.78	**<0.01**
Waist Circumference (cm)	95.1 ± 6.63	114.29 ± 12.39	**<0.01**
SBP (mmHg)	120.88 ± 13.97	127.82 ± 13.98	**<0.01**
DBP (mmHg)	78.4 ± 9.17	82.77 ± 9.75	**<0.01**
FPG (mg/dL)	86.86 ± 8.57	93.57 ± 13.34	**<0.01**
Insulin (μU/mL)	15.78 ± 8.73	26.27 ± 17.21	**<0.01**
Homa-IR	3.41 ± 1.94	6.16 ± 4.38	**<0.01**
HbA1c (%)	5.27 ± 0.36	5.42 ± 0.51	**<0.01**
Triglycerides (mg/dL)	69.28 ± 30.27	123.19 ± 63.01	**<0.01**
HDL Cholesterol (mg/dL)	54.43 ± 13.51	45.59 ± 10.91	**<0.01**
Total Cholesterol (mg/dL)	183.62 ± 36.92	196.41 ± 38.06	**<0.01**
LDL Cholesterol (mg/dL)	115.01 ± 31.62	127.28 ± 33.2	**<0.01**
Metabolic Syndrome (yes)	27 (7.40)	352 (41.20)	**<0.01** **^χ2^**
TSH (mU/L)	1.95 ± 1.21	1.98 ± 1.25	0.43
FT3 (pg/mL)	3.12 ± 0.42	3.16 ± 0.42	0.30
FT4 (pg/mL)	10.49 ± 1.41	10.63 ± 1.43	0.17
FT3/FT4 ratio	0.30 ± 0.05	0.30 ± 0.04	0.72
Vitamin D (ng/mL)	22.02 ± 8.01	19.5 ± 8.1	**<0.01**
Uric acid (mg/dL)	4.01 ± 1.09	5.01 ± 1.44	**<0.01**
AST (U/L)	19.15 ± 5.67	24.06 ± 9.8	**<0.01**
ALT (U/L)	33.67 ± 11.55	46.82 ± 23.08	**<0.01**
GGT (U/L)	20.92 ± 7.42	38.54 ± 27.07	**<0.01**

* Mann-Whitney test where not otherwise specified, ^χ2^ Chi squared test where not otherwise specified. Abbreviations: BMI (body mass index), SBP (systolic blood pressure), DBP (diastolic blood pressure), FPG (fasting plasma glucose), Homa-IR (homeostatic model assessment for insulin resistance), TSH (thyroid-stimulating hormone), FT3 (free triiodothyronine), FT4 (free thyroxine), AST (aspartate amino transferase), ALT (alanine amino transferase), GGT (γ-glutamyl transferase). Significance shown in bold.

**Table 2:** Description of the whole sample according to the Fatty Liver Index score after matching for age and sex (*N* = 732). All data are shown as mean ± SD (min to max) for continuous variables and as (%) for proportions.

	FLI < 60	FLI ≥ 60	*p* Value *
Prop. (%)	366 (50.00)	366 (50.00)	
Age (years)	37.62 ± 12.47	42.63 ± 13.69	**<0.01**
Sex			
*Females*	325 (88.80)	36 (9.80)	**<0.01**
*Males*	41 (11.20)	330 (90.20)	
BMI (Kg/m^2^)	28.85 ± 2.47	34.77 ± 5.80	**<0.01**
Smoking (Yes)	60 (16.90)	91 (25.90)	**0.03**
Fatty Liver index (%)	37.41 ± 14.62	87.24 ± 11.00	**<0.01**
Waist Circumference (cm)	95.10 ± 6.62	116.15 ± 12.61	**<0.01**
SBP (mmHg)	120.88 ± 13.96	132.28 ± 13.54	**<0.01**
DBP (mmHg)	78.39 ± 9.16	85.64 ± 9.793	**<0.01**
FBG (mg/dL)	86.86 ± 8.57	95.90 ± 14.53	**<0.01**
Insulin (μU/mL)	15.78 ± 8.72	27.61 ± 19.22	**<0.01**
Homa-IR	3.41 ± 1.94	6.61 ± 4.87	**<0.01**
HbA1c (%)	5.26 ± 0.36	5.45 ± 0.45	**<0.01**
Triglycerides (mg/dL)	69.28 ± 30.27	144.97 ± 71.65	**<0.01**
HDL Cholesterol (mg/dL)	54.43 ± 13.51	41.83 ± 10.30	**<0.01**
Total Cholesterol (mg/dL)	183.62 ± 36.92	198.95 ± 37.67	**<0.01**
LDL Cholesterol (mg/dL)	115.00 ± 31.62	129.57 ± 33.28	**<0.01**
Metabolic Syndrome (yes)	27 (7.40)	194 (53.70)	**<0.01**
TSH (mU/L)	1.94 ± 1.20	1.87 ± 1.18	0.49
FT3 (pg/mL)	3.11 ± 0.42	3.19 ± 0.421	**0.04**
FT4 (pg/mL)	10.49 ± 1.40	10.59 ± 1.41	0.47
FT3/FT4 ratio	0.30 ± 0.05	0.30 ± 0.05	0.39
Vitamin D (ng/mL)	22.01 ± 8.00	20.68 ± 8.23	0.08
Uric acid (mg/dL)	4.01 ± 1.08	5.81 ± 1.41	**<0.01**
AST (U/L)	19.15 ± 5.66	26.82 ± 10.63	**<0.01**
ALT (U/L)	33.66 ± 11.54	53.86 ± 26.20	**<0.01**
GGT (U/L)	20.92 ± 7.41	46.87 ± 28.88	**<0.01**

* Mann-Whitney test where not otherwise specified, ^χ2^ Chi squared test where not otherwise specified. Abbreviations: BMI (body mass index), SBP (systolic blood pressure), DBP (diastolic blood pressure), FBG (fasting blood glucose), Homa-IR (homeostatic model assessment for insulin resistance), TSH (thyroid-stimulating hormone), FT3 (free triiodothyronine), FT4 (free thyroxine), AST (aspartate amino transferase), ALT (alanine amino transferase), γ-glutamyl transferase (GGT). Significance shown in bold.

**Table 3:** Logistic regression model of a high risk of NAFLD according to the FLI score.

	*Raw model*	
	**Odds Ratio**	**CI 95%**
(Intercept)	0.275	0.091 to 0.831
FT3	1.506	1.064 to 2.131
	*Adjusted model*	
(Intercept)	0.002	0 to 0.144
FT3	1.557	0.784 to 3.089
HbA1c (%)	2.32	1.193 to 4.512

## Data Availability

The data presented in this study are available on request to the corresponding Author Roberta Zupo (email: roberta.zupo@irccsdebellis.it).
